# The Digital O&P Workshop

**DOI:** 10.33137/cpoj.v4i2.36349

**Published:** 2021-09-21

**Authors:** M Opitz, P. Fröhlingsdorf

**Affiliations:** MECURIS GmbH, Lindwurmstraße 11, 80337 München, Germany.

**Keywords:** Digital Transformation, Central Fab, Digital Process Chains, FDA, Custom-Made Device, Prosthetic Feet, Orthotic Brace, Quality Assurance, Additive Manufacturing, Trends

## Abstract

Digitalisation is the megatrend in healthcare, not only since the pandemic. We are two European digital health experts and industry leaders in the field of orthotics and prosthetics (O&P) and in this article we explored what are the underlying trends driving the adoption of digitalisation for customisation of prosthetics & orthotics. We showed that several trends in 3D image capture (input step), 3D modelling (processing step) and 3D printing (output step) currently converge and thus fuel the rapid transformation of the O&P industry. In short outlooks, we rated the probability and timing of adoption rates across the upcoming couple of years. We furthermore reviewed the impact of boundary conditions set by regulators as well as the reimbursement system. Towards the end of this article, we outlined a digital scenario of the near future by following around an orthotist during her work. We finished with a call-to-action targeting regulators, payors, prosthetists/orthotists, and patients to enable such a desirable future.

## CONVERGING TRENDS DRASTICALLY DIGITALIZE O&P WORKSHOPS

Digitalisation alone is just a meaningless buzzword. Digitalisation is not a trend. In the scientific discipline of future studies,^1^ digitalisation would be tagged as a megatrend. Megatrends span several sub phenomena we call (tech) trends like 3D scanning, 3D modelling and 3D printing.

Several of these trends currently converge and speed up change in orthotics & prosthetics (O&P) workshops up to a so-called tipping point. This means from a certain point in time or rather a rate of adoption, change fuels itself in a vicious circle and cannot be stopped anymore. Popular examples are “flatten the curve” efforts versus the Covid pandemic,^2^ “network effects” in social networks like Facebook,^3^ global warming tipping points in climate change.^4^

In addition, Covid-19 broke up traditional ways of working. Such unforeseen events are called wildcards. Wildcards drastically reduce the resistance to behavioural change and establish “new normals”. After such an event, things tend to snap “back to normal”. But, the longer the current crisis continues, the more routines have already changed permanently.

The likelihood of future scenarios is strongly influenced by boundary conditions that are unlikely to change as rapidly as the fundamental needs of the end users, the regulatory framework and reimbursement.

**Figure 1:**
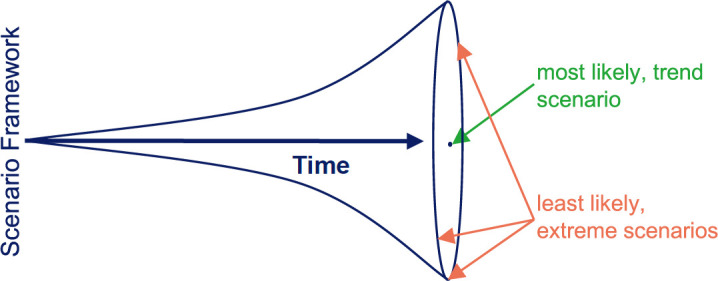
Framework for classifying scenarios based on their probabilities.

It is impossible to say what the future will look like. We can only describe scenarios and estimate their probabilities (**Figure 1**). All probable scenarios currently foresee a fast-paced change towards a more digital manufacturing process. There is no “if”, just “when”. That means, all those who do not want to “suffer change” but instead create the future of O&P, now have to openly debate the “how”. In particular, how do we want the future of assistive device production to look like? Is there a scenario in which all healthcare customers benefit: end users, providers and payors?

This paper focuses on the O&P workshops and, in the first section, provides an overview of the trends around digitalisation that are about to converge and potential future adoption scenarios. The next section reviews how much boundary conditions prohibit or foster change in O&P care. In the final section, the impact of the Covid wildcard will be assessed and we describe a desirable scenario that works as a north star for end users, providers and payors. We conclude with a call to action to all stakeholders, summarizing the next steps to be taken to increase the likeliness of this favourable scenario.

## CONVERGING 3D TECHNOLOGY TRENDS

In digitizing the laboratory, we have to look at the three major steps of the process:

1. Input (e.g. enabled by 3D scanning)
2. Processing ( e.g. enabled by 3D modelling software)
3. Output (e.g. enabled by 3D printing or CNC milling)

### Input - Smartphone 3D scanning apps validated for O&P lead to data explosion

**Figure 2:**
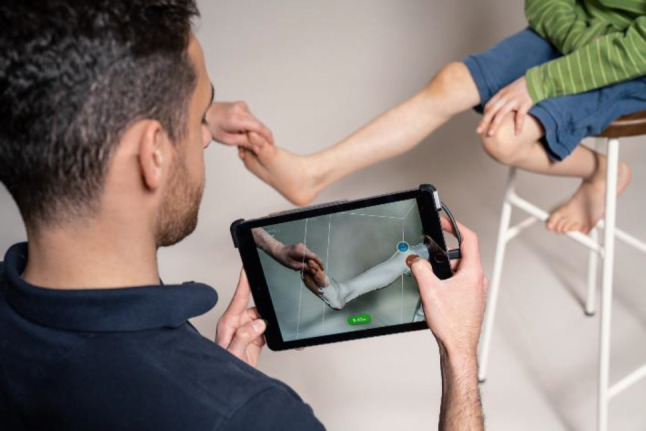
Use of a simple, tablet-based 3D scanner. Persons in image have given informed consent to publication.

3D scanner adoption varies a lot by country. Some countries like France with few O&P clinics serve large areas with central fabrication done by unskilled workers and, with favourable reimbursement systems, have almost full 3D scanner adoption. In Germany, adoption is around 64.4%, with another 22% planning to use 3D scanners soon (**Figure 2**), according to a 2017/18 survey among 118 German CPOs.^5^ The US and Canada are surprisingly non-digital in this category.

As of now, almost every CPO practicing in Canada or the US has a 3D scanner in their jeans pocket. Most current smartphones support 3D scanning but most generic 3D scanning apps^6^ for both Android and iOS, have not been validated for patient scanning. With the advent of more and more scanning apps specifically targeting O&P, validating patient scanning is beginning to happen,^7,8^ usability and scanning quality are being greatly increased and adoption rates are growing fast. Since the iPhone X,^8^ most models currently in use support these O&P scanning apps to an accuracy of ±1mm.

In considering this, the tipping point for widespread adoption of ready-to-scan smartphones already lies in the past. The only question is, if the future follows a scenario with 3D scanning apps going mainstream in O&P in 2021 or 2022, or if some unforeseen event still changes this trajectory.

### Processing - Workflow-based Software-as-a-Service (SaaS) modeling software pulverises entry barriers

3D modelling software has been used in all larger O&P workshops for several decades.^9^ However, two major factors prohibited widespread adoption in smaller workshops:

1. The high cost of $20'000 or $30'000 for a single license. Annual maintenance fees add to the initial cost.
2. High expertise in CAD design is needed. In many countries this is not threaded into the P&O educational curriculum and post-graduate training in these skills is costly, time-consuming and results in a lower level of competence than if it were integrated into the core P&O curriculum.

Additional minor factors include too few software updates, issues due to licenses being fixed to a single machine, high demand of processing power in a local machine, the requirement for specific tools like CAD pens, etc.

These issues are not exclusive to the O&P industry. In other industry areas, they already have been solved with modern CAD tools.

The high initial cost barrier was overcome by SaaS offerings. Every CPO uses SaaS in their private life, e.g. through streaming services like Netflix or Spotify. The accountability of monthly fees for products is also increased thanks to that. On the technical side, subscription, web-based software that requires a monthly fee allows for access from any device, paving the way not only for SaaS, but also for faster updates and bug fixes, while reducing requirements for local hardware by allowing for processing directly in the cloud.

**Figure 3:**
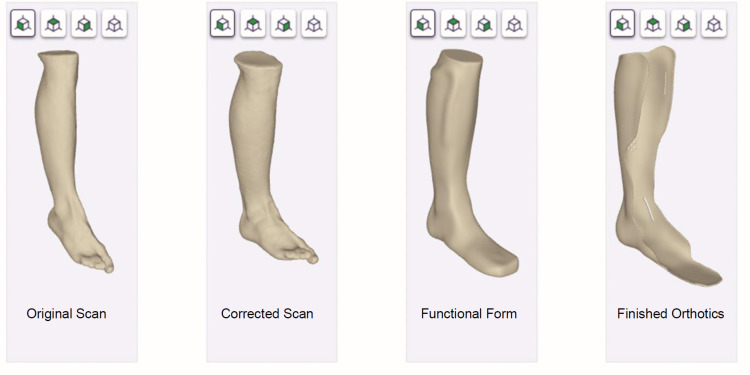
Focus on usability: clean and intuitive user interface and follows the “natural”, manual way CPOs are used to, with only a few tools that are required for a certain process step being visible.

Modern software focuses on usability. For widespread adoption, a clean and intuitive user interface is key (**Figure 3**). This runs counter to classic CAD software offering up several dozen tools in one interface. Workflow-based software, in contrast, follows the “natural”, manual way CPOs are used to and expect (**Figure 3**).

This modern software offers only the few really necessary tools required for a certain process step. FAQs and feedback/help tools are also specific for every step.^10^ Thus, little to no training is required and providers are “trained on the job”. Again, cloud technology enables powerful smart features like an assisted posture correction of a scan or suggestions for functional form creation using machine learning.^10^

Right now, such tools are primarily being offered to providers by startups. Most focus on one application area, e.g. sockets^11^ or wrist braces.^12,13^ Only a few enable several applications such as a classic CAD tool for the orthotics and prosthetics side.^10^ However, the two leading manufacturers have also started in this direction, with Ottobock investing in several startups^14^ and now developing their own workflow-based orthotics modelling software and Össur, this year, announcing the purchase of parts of the socket modelling software from Standard Cyborg.^15^

The 7-digit investments made by dominant market players like Ottobock and Össur into process-based modeling software and the variety of new ideas by startups clearly indicate that the market is already tipping in this direction. Fueled by the abundance of available 3D scanning data the remaining question is not if, but when 3D modelling software goes mainstream in O&P: 2022 or 2023 or 2024.

### Output - Proliferating 3D printing service allows direct print of final design

**Figure 4:**
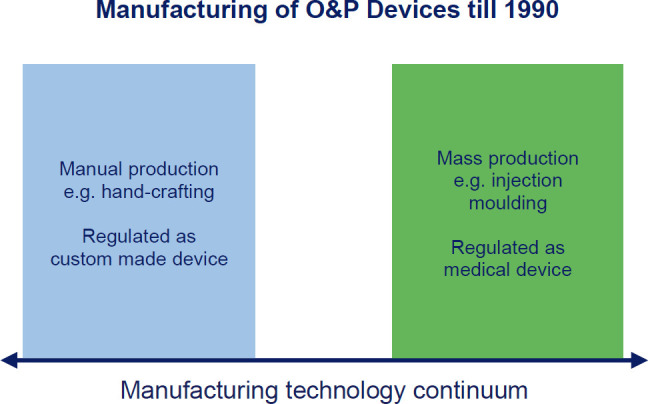
Two predominant ways of manufacturing dominated global production: Injection moulding for mass production and manual hand crafting for custom products, with differing, clearly separated regulatory requirements.

Today, two ways of manufacturing dominate global production: Injection moulding for mass production and manual hand crafting for custom products (**Figure 4**). But, over the last few decades, mass customization enabled by the advent of new manufacturing technologies has begun to bridge the chasm between those two extremes. Most relevant for O&P are computerized numerical control (CNC) milling and, more recently, additive manufacturing (AM), often referred to as 3D printing.

Automation offers the highest leverage with large quantities, even for individual care. Therefore, CNC milling machines were first and most successfully adopted in high volume individual care industries, as in dental or orthopedic insoles. However, severe restrictions on the complexity of the design limited the use of CNC milling machines. This was the case with the manufacturing of hearing aids, which could not be widely manufactured CNC milling, other than for some of the internal parts. Therefore, the hearing aid industry was able to leapfrog CNC milling and went directly to AM showing a concerted progress within a few years. Using this strategy, the hearing aid industry was able to move to directly producing the final product - without any mold or pre-product.

By lowering costs per part, growing build volumes and increasing process stability, AM has now reached maturity for O&P applications beyond orthopedic insoles. This finally enabled a fully digital process chain. Before, it was aborted with the functional form that is milled and then used as a negative for thermo forming of an orthosis. This also meant a “dirty” workshop was still necessary. Now, the (negative) functional form can be digitally “wrapped” with a (positive) final orthosis (or socket) design. This design, in turn, is directly manufactured using AM. Digitalising this last step saves a lot of working time for the provider, e.g. 3.7h for a foot orthosis.^16^ For larger providers, this quickly scales into a substantial economical advantage.

The clear downside with AM is the exponential cost scaling with increasing build volume. Therefore, it is less a question if prosthetics or orthotics are economical to print. The question rather depends on the build size of the application. Small O&P devices especially in pediatrics are already much cheaper to print compared to manual production, often even milling. This includes pediatric prosthetic feet, AFOs, DAFOs, night splints, wrist orthotics and baby helmets. For larger build volumes, CNC milling however remains the mass customization method of choice. This is true especially for large bracings for adolescents or adults that have to endure high stress and have to be built in one part, e.g. corsets.

The paradigm shifts from subtractive to additive and also more unique selling propositions (USP): Less waste. In times of rising environmental awareness, saving 1.5kg of waste and 1.6kg CO2 equivalent in the manufacturing of a 0.5kg foot orthosis by using AM instead of hot forming is important to many end users.^16^

A more long-term scenario includes the widespread shift in thinking from recreating traditional, subtractively manufactured devices with 3D printing towards more natural, generative designs.^17^ This shift in mindset rivals another scenario, in which a widespread availability of more materials, especially those known in the O&P space, lead towards another jump in adoption.

**Figure 5:**
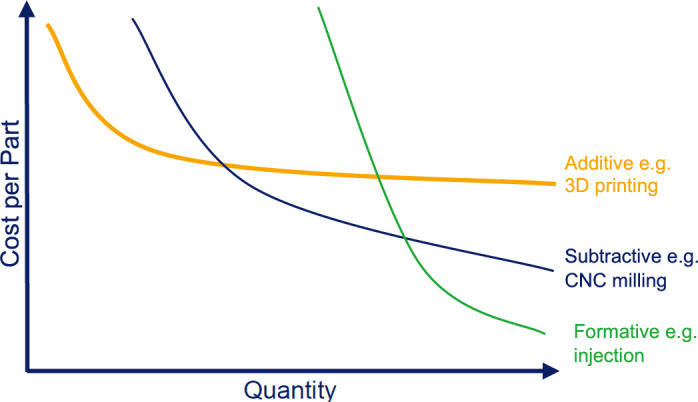
Schematic comparison of manufacturing technologies by cost-per-part and quantity of produced parts.

In conclusion, the 7-digit investments made by dominant market players like Hanger and Ottobock as well as national purchasing associations e.g. in Germany^18^ into industrial 3D printers and post processing machine parks clearly indicate that they expect additive manufacturing to gain a 2-digit market share in the very near future. Most scenarios predict 3D printing as one more production tool and not the dominant technology overall, likely on par with CNC milling, injection moulding (**Figure 5**) and, yes, some minor modifications still being done by hand.

Due to the aforementioned high investment cost, most providers except the very large players like Pohlig in Germany or EastPoint^19^ in the US will either rely on a dominant OEM for 3D printing, together with their peers co-fund a 3D printer in a central fab^20^ e.g. within the framework of a purchasing association, or rely on a “neutral” 3rd party, the mushrooming number of 3D printing service bureaus. The scenarios for the future digital supply chains vary a lot by country and the competitive situation. It is likely that after the current adoption wave fueled by O&P-ready printing hubs and sinking costs, there will be another spike of adoption in a few years with the availability of more materials or the understanding of the new freedom of design enabled by AM.

## BOUNDARY CONDITIONS

### Regulation driven by FDA separates Custom + Patient-Matched Device

Now that the fully digital process chains are capturing more and more medical markets, regulators are adjusting the framework in which manufacturers are allowed to operate. Former regulations often did not cover these areas, or they allowed too much or too little legroom for manufacturers for patient safety. This caused a flurry of updated or new definitions and guidelines by national and international authorities. Most productive has been the FDA with advanced regulations for AM of medical devices,^21^ software as a medical device (SaMD), in silico-trials (virtual testing) and more. The FDA has worked alongside other national regulators in the International Medical Device Regulators Forum (IMDRF) to assure a globally consistent definition for new terms like SaMD or patient-matched medical device.

**Figure 6:**
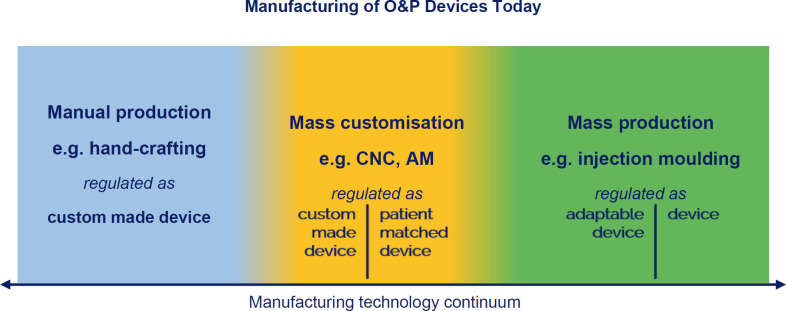
Regulatory Overview by production technology: nowadays, there is no clear-cut border anymore between custom-made and mass-produced devices, as mass-customisation has filled the gap. Regulation cannot differentiate by production technology anymore, but needs to look at who is in charge of modelling and manufacturing.

The IMDRF has also filled the regulatory chasm between manual and mass manufacturing (**Figure 6**), defining sub-types for personalised devices and providing technology examples for mass customisation like 3D printing: “It is now possible to produce medical devices, which are individualised e.g. by additive manufacturing methods (3D printing) based on patient CT scans, on a commercial rather than a manual scale. The original GHTF documentation does not adequately address such devices.”^22^ Further motivation was to make sure that the regulatory reliefs granted to custom made device manufacturers are not abused by industrial manufacturers to produce large quantities: “In other jurisdictions, the derogations were established with the intention that the number of custom-made devices would necessarily be small, as they could only be used in special cases.”^22^ The FDA explicitly limits the production of custom made devices "to no more than five units per year of a particular medical device".^23^

Today, the IMDRF distinguishes between 3 subclasses of personalised medical devices:^22^

1. Custom-made products: A custom-made medical device on the written order of an authorised (medical) professional. This professional, on his own responsibility, gives the medical device specific design features, even if the design was developed with a manufacturer. In a nutshell, the provider controls the design step. It does not matter:
  1.1. if the provider uses a 3rd party software to do so.
  1.2. if the manufacturing is carried out by a 3rd party.
  1.3. whether the device is hand-crafted or 3D printed.
2. Patient-matched medical device: It is designed and manufactured under the responsibility of a manufacturer, even if the design was developed in consultation with an authorised healthcare professional. In a nutshell, a 3rd party manufacturer controls the design step. It does not matter:
  2.1. if the provider supports with initial patient data or other design inputs.
  A practical example are plaster casts and/or 3D scan of a patient anatomy that is sent to a manufacturer who adapts a digital orthosis model to fit this patient anatomy and then returns a patient-specific manufactured orthosis.
3. Adaptable medical device: A mass-produced product that is adapted, adjusted, assembled or shaped at the point of care, in accordance with the manufacturer's validated instructions. It does not matter:
  whether the healthcare professional or the patient him/herself adapts or adjusts the device.

A practical example are orthoses that are adapted to the patient through thermoforming, and/or are adjusted by the patient.

This means that the globally valid definition distinguishes a patient-specific medical device from a custom-made product primarily because the design step is within the responsibility of the manufacturer, not the provider. The Medical Device Coordination Group (MDCG), a group formed on request of the European Commission representing all member states, has just now adopted this definition in their MDCG 2021-03 guideline on custom-made devices.^24^

The trend scenario will thus see a continuation of new guidelines^25^ or updates of current ones, especially in the EU that was so far busy releasing the MDR. A harmonization of global definitions as national directives like the MDR or guidelines (EU, Australia, FDA) will probably take up to 5 years. Canada also has not yet updated their regulations^26^ and guidelines^27^ to account for mass customised devices. However, it is likely that the EU and FDA have adopted the IMDRF definitions, most other countries follow in their wake, which hopefully leads to a more unified regulatory landscape. More long-term, regulators might go after manufacturers misusing the custom device reliefs. It seems likely though that O&P manufacturers will benefit from a grace period compared to medical manufacturers in higher risk classes that pose a higher risk to patient safety. Providers will always benefit from these reliefs. One scenario might be a care provider suing a large manufacturer that tries to grab a larger part of the value chain by not only offering modelling software or manufacturing services, but also internalising the design control. An alternative scenario might be a lawsuit by a competing manufacturer.

### Reimbursement's major Impact could Stifle or Speed up Digitalisation

Payors also want to benefit from this innovative process. The megatrend of ageing population in countries of the Global North and thus more patients per capita forces payors to constantly lower reimbursement rates to rebalance overall healthcare costs. Growing margins for providers that have adopted a digital process chain increases the likelihood for future scenarios in which payors will once more target O&P reimbursement levels. These scenarios will force manual manufacturing providers to either switch or shut down. The most likely scenario drives all providers to digitize their process of care giving in a short time frame, all but a few highly specialised providers that operate in a niche like pediatrics or para athletes and/or providers that increase their margin by out-of-pocket payments. However, since the year 2000, out-of-pocket payments have been constantly in decline in the US and Canada.^28^

Another long-term benefit for both regulators and payors is the perfect measurability of digital processes. Providers will gain unprecedented quantitative insights into their manufacturing process. From scanning to printing, every step is monitored and evaluated. This enables a continuous improvement process as adopted in most industries by now,^29^ leading not only to less errors for providers, but also improved results for end users. Less complications and better patient outcomes are also the goals of regulators and payors, reducing secondary costs like refittings, rehabilitation, and product replacements. In an unlikely scenario, payors therefore use their foresight to pay extra for custom made devices that were additively manufactured to drastically reduce adoption time and thus reap the benefits much faster. One example of this was the increase in reimbursement rates for O&P devices reinforced with carbon fibre for increased durability as the decisive factor for the sudden widespread adoption across all providers.

### CONCLUSION

#### Covid Wildcard

Due to its long duration and global impact for all citizens, the Covid crisis has a tremendous impact, even compared to other wildcard events like 9/11 or the Fukushima reactor catastrophe. This means the likelihood of future scenarios requiring more drastic behaviour changes increases, the longer this crisis endures.

For end users, the sudden advent of telehealth will increase the demand for less physical appointments also in O&P care. This was clearly unthinkable at the beginning of 2020. Taking a more active and informed approach towards healthcare will also increase demand towards providers to be treated accordingly.

Providers, in turn, would not have expected to work in home offices in O&P care. As most CPOs do not have a plaster cast room at home, digital and distributed workshops suddenly became very important. These new tools are not part of the current reimbursement landscape.

Therefore, payors that have already been quick to provide temporary reliefs like waiving or accepting digital prescriptions or bonuses for protective equipment will also have to address such requests. Tremendous costs on the one side were partly offset by drastically reduced spending for elective surgery and other healthcare treatments, evidently showing that healthcare spending is much less fixed than assumed for decades.

#### Desirable Scenario for End Users, Providers and Payors

Two paradigms seem constant for most future scenarios. First, the ageing population, which in turn leads to decreasing reimbursement rates by payors and thus increasing cost pressure for providers or out-of-the-pocket spending for end users. A second constant seems to be that digitalization as a technology will not replace human interaction and improve healthcare on its own, but empower all stakeholders with new tools and thus change how care is delivered in the future. Based on these assumptions, we will draw a likely and desirable trend scenario for the O&P care of the new future in following:

In 2022, Tim needs a new AFO as his old one broke yesterday during work. Due to his impaired mobility, he schedules a quick video call with his orthotist Emma. After a quick look at the orthosis, Emma decides it is beyond even temporary repair, snaps a few pictures and files a complaint so the next AFOs will have a sturdier design for Tim. The health insurance is informed, too.

The next day, Emma visits Tim at home to collect the broken orthosis. As always, she takes inventory on joint mobility and muscle status and of course skin irritations, too. Thanks to this checkup, Emma identifies a recent pressure ulcer on the left side of his ankle, unrelated to the defect. She finally scans his foot with her mobile phone. Tim picks green and yellow as colors for his new AFO and a wavy pattern for the backside. When he is fully happy with the 3D preview, he adds his initials on the design - just like an artist would do after expressing himself. With the technical information quickly collected, Emma uses the rest of her time to understand Tim's recently changing activity profile and builds their relationship of trust.

**Figure 7:**
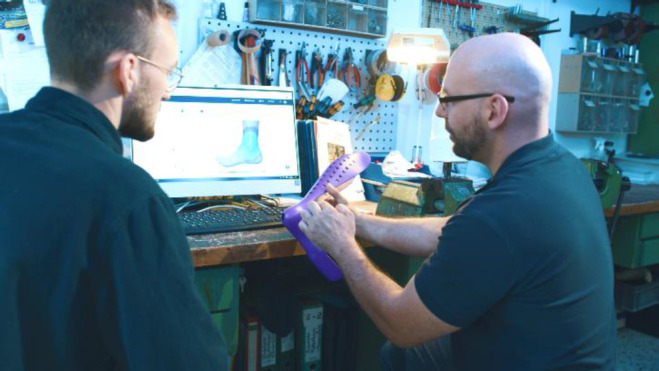
Review of design changes using digital tools. Persons in image have given informed consent to publication.

After visiting several more of her patients, Emma receives approval to create a new orthosis from the payor thanks to her digital defect report. Despite the premature defect, they are happy that Tim is evidently very active and thus lowers the risk of higher follow up costs if his mobility decreases. Emma uses 30 minutes in the afternoon to configure Tim's new AFO on her workstation at home. She reloads the previous AFO design for Tim and asks the software to resize it based on today's 3D scan. She then reinforces the defect area and reshapes the pressure point so she can add more padding for Tim's ankle. She then runs a quick computer simulation to assure the design changes have not increased the risk of failure or impaired the functionality of the device.^30,31^ She also invites colleagues at her workshop, Bob and Tommy, to review her design changes digitally (**Figure 7**).

When Emma arrives in her workshop the next day, she receives a notification that due to an anomaly during the printing process, the material of the printed shell has slightly darkened. It seems to her like coloring issues in orthosis manufacturing have “successfully” transitioned from manual to digital manufacturing. Thus, she quickly sends a new 3D preview to Tim using a darker orange instead of the bright yellow so the darkened material does not shine through. Tim is very happy with the new color. However, he had second thoughts about “signing off” his AFO and asks Emma to not color his initials too prominently, as he is afraid it might look too cocky.

The next day, Emma receives the colored shell and quickly inspects the quality and documents it with a few photos. She then adds the straps and padding, especially on the left side of the ankle. In the afternoon, she visits several end users, including Tim. He tries out the new AFO and is impressed with the consistent, perfect fit and the small improvement of the padding. Emma captures a few videos of his gait to document the great functionality for her colleagues and the payor. Tim also answers a quick satisfaction survey, stressing that he is not only very happy with the result of his new orthosis, but also the effortless complaint procedure with the provider and the payor. Not only is he mobile again, but also a proud co-creator of his personalised assistive device.

## CALL TO ACTION

To all end users: As described above, you can now take part in the aesthetic design of your custom patient aid. This is just one example of how digitalisation empowers you to become the co-creators of your own devices, together with your trusted provider.

To providers: If you care more about patient outcomes than the manual craftsmanship in your workshop, digitalisation enables you to shift your focus while increasing customer satisfaction and productivity. No software or 3D scanner is going to replace the unique bond you have with your clients, but if you prefer tinkering over caring for patients, payors will make your life much more difficult in the future.

To payors: To reap the true benefits of digitalisation in O&P care, a small reimbursement incentive drastically increases adoption time, thus lowering long-term post-treatment costs thanks to high and consistent health outcomes.

To regulators: Despite not being a healthcare customer, you have the power to guide efforts for improved patient outcomes without harming innovation. The main need is not for additional regulation, but further, more tangible guidelines, ideally globally harmonised. This stretches from SaMD to mass customisation and fabrication to prevent the misuse of custom made device reliefs.

## DECLARATION OF CONFLICTING INTERESTS

The authors are members of the executive management team and/or shareholders of the Munich-based company Mecuris. The Mecuris Solution Platform offered by Mecuris contains digital tools as described in the article.

## SOURCES OF SUPPORT

Parts of this article have been enabled through the research project SIGMA3D. This includes the cited works on regulation of custom-made devices and the MDR of Ann-Kathrin Carl from University of Applied Science Münster. Mecuris is the project leader and the project is funded by the German Federal Ministry of Education and Research under the funding code 16SV8386. The responsibility for the content of this publication lies with the authors.

## AUTHOR SCIENTIFIC BIOGRAPHY

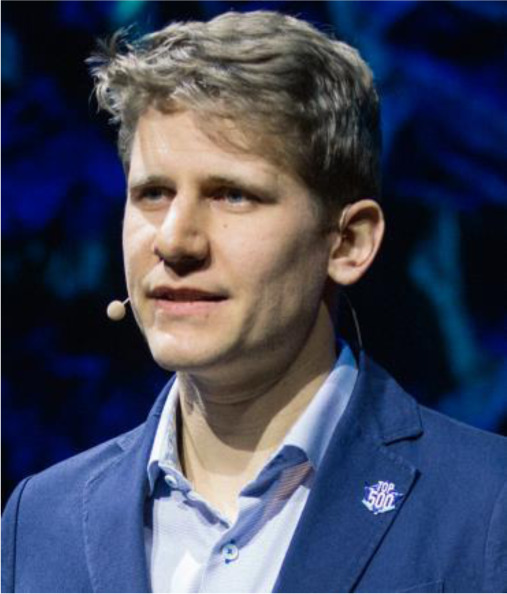



Before starting several medtech ventures, **Manuel Opitz** studied industrial & bioengineering at RWTH Aachen and Trinity College Dublin (MSc). After first experiences in innovation and operations management in Germany, China and Switzerland, he finished postgraduate studies in economics at the trinational CDI in Paris (MBA). Afterwards, he focused on medical technologies as a patent broker and startup consultant. He successfully raised funds for three different medtech ventures. As co-founder of Mecuris (CEO & COO), he analyses orthopaedic manufacturing processes to unlock digitalisation potential for orthotists/prosthetists and patients. With his newest venture (CEO), he aims at reducing diagnostic errors and patient visits using AI software as a second opinion for doctors. As a regular speaker at healthcare conferences (OTWorld, ISPO, …), innovation (TEDx) and startup conferences, he works on bridging the gap between digital technologies and the healthcare system. He is co-author of an industry guideline on “digital process chains in medical technology” and contributed to books on 3D printing as well as digital business models in the healthcare sector.

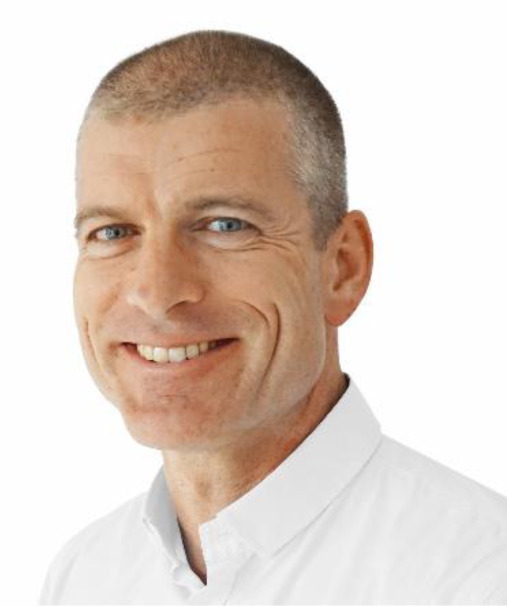



Master craftsman and certified prosthetist & orthotist **Peter Fröhlingsdorf** gained hands-on experience in patient care with his own handicraft production for 10 years before he successfully took over the responsibility for setting up a central production for orthopaedic and orthopaedic shoe technology aids in one of the largest medical supply stores in Germany. There he later expanded and developed the area of children's and youth care as managing director. Further career stages include 5 years of sales experience with an Icelandic prosthesis manufacturer and setting up a European sales hub and force for assistant robots of a Canadian manufacturer. Peter Fröhlingsdorf has been CEO of Mecuris GmbH since January 2020. He is a regular lecturer at the Federal College for Orthopaedic Technology in Dortmund, OTWorld, RehaKind, Focus CP, Accident Insurance Association and more. He is also 2nd chairman and treasurer of Cerebral Palsy Network e.V. and is passionate about innovation in orthopaedic technology.
